# Onset of the spring bloom in the northwestern Mediterranean Sea: influence of environmental pulse events on the *in situ* hourly-scale dynamics of the phytoplankton community structure

**DOI:** 10.3389/fmicb.2014.00387

**Published:** 2014-08-12

**Authors:** Melilotus Thyssen, Gerald J. Grégori, Jean-Michel Grisoni, Maria Luiza Pedrotti, Laure Mousseau, Luis F. Artigas, Sophie Marro, Nicole Garcia, Ornella Passafiume, Michel J. Denis

**Affiliations:** ^1^CNRS/INSU, IRD, Mediterranean Institute of Oceanography, Aix Marseille UniversitéMarseille, France; ^2^Sorbonne Universités, UPMC Univ. Paris 06, UMR 7093, LOV, Observatoire OcéanologiqueVillefranche-sur-Mer, France; ^3^CNRS, UMR 7093, LOV, Observatoire OcéanologiqueVillefranche-sur-Mer, France; ^4^Laboratoire d'Océanologie et Géosciences, Univ. du Littoral Côte d'Opale, CNRS, UMR8187Wimereux, France

**Keywords:** Coastal Mediterranean Sea, spring bloom, *in situ*, phytoplankton, remotely controlled flow cytometry, diel variations, cell cycle

## Abstract

Most of phytoplankton influence is barely understood at the sub meso scale and daily scale because of the lack of means to simultaneously assess phytoplankton functionality, dynamics and community structure. For a few years now, it has been possible to address this objective with an automated *in situ* high frequency sampling strategy. In order to study the influence of environmental short-term events (nutrients, wind speed, precipitation, solar radiation, temperature, and salinity) on the onset of the phytoplankton bloom in the oligotrophic Bay of Villefranche-sur-Mer (NW Mediterranean Sea), a fully remotely controlled automated flow cytometer (CytoSense) was deployed on a solar-powered platform (EOL buoy, CNRS-Mobilis). The CytoSense carried out single-cell analyses on particles (1–800 μm in width, up to several mm in length), recording optical pulse shapes when analyzing several cm^3^. Samples were taken every 2 h in the surface waters during 2 months. Up to 6 phytoplankton clusters were resolved based on their optical properties (PicoFLO, Picoeukaryotes, Nanophytoplankton, Microphytoplankton, HighSWS, HighFLO). Three main abundance pulses involving the 6 phytoplankton groups monitored indicated that the spring bloom not only depends on light and water column stability, but also on short-term events such as wind events and precipitation followed by nutrient pulses. Wind and precipitation were also determinant in the collapse of the clusters' abundances. These events occurred within a couple of days, and phytoplankton abundance reacted within days. The third abundance pulse could be considered as the spring bloom commonly observed in the area. The high frequency data-set made it possible to study the phytoplankton cell cycle based on daily cycles of forward scatter and abundance. The combination of daily cell cycle, abundance trends and environmental pulses will open the way to the study of phytoplankton short-term reactivity to environmental conditions.

## Introduction

Phytoplankton plays a major role in marine ecosystems as it is the main primary producer in the euphotic layer. Its production in coastal areas can represent up to 30% of the global oceanic primary production (Gattuso et al., 1998), and the inputs of coastal production to the open sea can drive high productivity in near-shore areas (Robinson and Brink, 2005). The uncertainty regarding phytoplankton production estimates is largely due to under-observation. Phytoplankton communities are highly diverse and were shown to respond to environmental changes at the scale of the hour (Jacquet et al., 2002; Thyssen et al., 2008b; Lefort and Gasol, 2013). This fast response capacity depends principally on the growth rate of some pico and nanophytoplankton species. They display daily cyclic variations of abundance due to the combination of synchronized cell cycles and losses (grazing, viral lysis, sinking), though some very high increases in abundance have been observed after intense and sporadic environmental changes (Thyssen et al., 2008b; Dugenne et al., this issue). These high increases in abundance could not be fully explained by the doubling of the population, which calls for faster cell cycles and/or higher growth rates under specific conditions. Depending on the sampling strategy, sampling at one time or another may completely change the interpretation of the phytoplankton community structural changes (Dubelaar et al., 2004), thereby leading to misunderstanding and underestimating the role of phytoplankton production in the ecological and biogeochemical status of the studied area (Taylor and Howes, 1994; Riser and Johnson, 2008). Pulse perturbations such as storms and wind events have a large influence on the exported production. They may induce fast responses of nanophytoplankton (Lomas et al., 2009), or a burst of abundance of picophytoplankton liable to form aggregates heavy enough to sink (Richardson and Jackson, 2007; Lomas and Moran, 2011). Furthermore, autotrophic picoeukaryotes were shown to be of importance at the onset of the spring bloom (Calvo-Diaz et al., 2004), and it is hypothesized that any change has the potential to affect the usual succession pattern of the spring bloom, and consequently the food web structure itself. High-frequency sampling of phytoplankton is thus a fundamental requirement to record these events when they occur. Moreover, since phytoplankton species exhibit different biogeochemical capacities, one must take into consideration functional phytoplankton diversity and not be content with a global estimation of biomass based on bulk chlorophyll content, size classes, or low frequency taxonomical features data-sets (Quéré et al., 2005). There are now several *in situ* technologies capable of delivering such information. As an example, the automated flow cytometer (Dubelaar and Gerritzen, 2000; Olson et al., 2003) is able to carry out single-cell analysis and to discriminate functional groups at the hourly scale, while the Environmental Sample Processor is capable of high-frequency species recognition using targeted probes (Greenfield et al., 2006).

The Mediterranean ecosystem, considered as a biodiversity hot spot (Smith et al., 2001), is foreseen as one of the most sensitive areas as far as the forthcoming climate change is concerned (Giorgi, 2006). Increase in temperature will lead to dry and windy periods (see references in Durrieu de Madron et al., 2011). Stratification will be counter-balanced by water mixing, with amplitudes that should be higher than the ones currently observed. In addition, atmospheric pollution (induced by human activities) and natural dust deposits will affect surface marine ecosystems (Lenes et al., 2001; Pulido-Villena et al., 2008). There is a need to observe the influence of such events on biological and physical long time series data-sets, as it is the case since, 1957 in the Bay of Villefranche-sur-Mer at Point B. The trophic status in the Bay of Villefranche-sur-Mer (NW Mediterranean Sea) is mostly dominated by the microbial loop (Rassoulzadegan and Sheldon, 1986) and microphytoplankton abundance is considered to be low compared to other northwestern Mediterranean bays (Ferrier-Pagès and Rassoulzadegan, 1994). Pico- and nanophytoplankton cells were shown to exhibit the fastest responses to environmental changes, and they may drive the efficiency of the production in coastal oligotrophic areas, partially sustaining open sea production. In this oligotrophic area, pico- and nanophytoplankton are mainly consumed by tintinnids (Rassoulzadegan et al., 1988; Bernard and Rassoulzadegan, 1993), which play a major role in linking up the microbial loop and the classical food web (Sherr and Sherr, 2000). Previous observations in the Bay of Villefranche-sur-Mer evidenced different patterns for the onset of the “spring” phytoplankton bloom. According to the most common pattern, the spring phytoplankton bloom takes place in February-March and is dominated by pico-nanophytoplankton. It is followed in May by a diatom/dinoflagellate-dominated bloom (Gomez and Gorsky, 2003). The common succession pattern observed is pico-nano/diatoms/dinoflagellates. However, in some years, the microphytoplankton spring bloom is weak because the balance between water column stabilization and nutrients availability is not reached (Bustillos-Guzman et al., 1995; Gomez and Gorsky, 2003).

Long time series have shown that phytoplankton resilience to environmental changes (especially temperature) is strong in very nutritive areas such as the North Sea (Wiltshire et al., 2008) and in the oligotrophic waters of the northwestern Mediterranean Sea (Norberg and DeAngelis, 1997). However, the effect of meteorological and hydrological pulse events on phytoplankton community structure and dynamics occurs at a scale that is poorly detected by traditional weekly or monthly sampling time series. It may cause the spatial displacement of populations due to hydrodynamics, especially after strong wind events (Furuya et al., 1993), but it may also affect growth rate through changes in the cell cycle and grazing pressure (Lefort and Gasol, 2013).

This study aimed to better understand the influence of meteorological and hydrological changes on the onset of the phytoplankton bloom in the Bay of Villefranche-sur-Mer. In this objective, we here used for the first time an automated and remotely-operated flow cytometer fitted inside an autonomous solar-powered buoy moored in the bay. The automated flow cytometer records the optical signals (fluorescence and light scatter pulse shapes) generated by every particle (cell) crossing a laser beam. By combining high-frequency sampling and the flow cytometry analysis of particles up to 800 μm in width and a few mm in length, involving volumes of several cm^3^, we were able to meet the above-mentioned requirements for optimal phytoplankton observation since samples were analyzed almost every 2 h over more than 2 months. The abundance dynamics and cellular optical properties reflecting the cell cycle of the different phytoplankton clusters are presented and discussed in the light of the co-occurring hydrological and meteorological conditions.

## Materials and methods

### Study site

**Figure 1:**
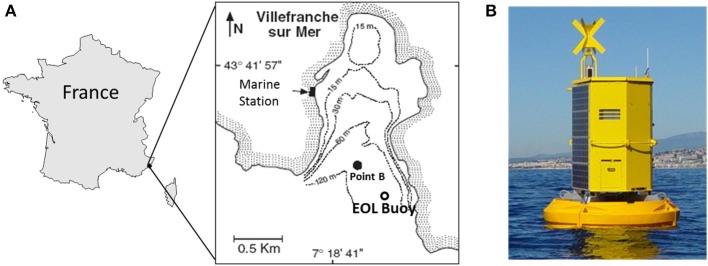
**(A)** Location of the study site in the Bay of Villefranche-sur-Mer (northwestern Mediterranean Sea). Point B corresponds to the SOMLIT (*Service d'Observation en Milieu Littoral*) sampling point where samples are collected weekly. **(B)** The automated CytoSense flow cytometer was fitted inside the EOL buoy.

The *in situ* sampling was conducted in, 2012 from the EOL-Environnement Observable Littoral (CNRS-Mobilis) buoy moored in the Bay of Villefranche-sur-mer (43.682°N, 7.319°E; Figure 1A) from January 24 to April 6. The EOL buoy (Figure 1B) is located 355 m away from the SOMLIT (Coastal observation service) monitoring station labeled Point B (43.686°N, 7.316°E; Figure 1A). The depth of the water column is ~100 m at both sites.

### The EOL buoy

The EOL buoy (Figure 1B) was developed by the Oceanological Observatory of Villefranche-sur-Mer (CNRS-UPMC) and is commercialized by Mobilis. It is dedicated to the observation of human activity impacts (pollution, urbanization, tourism, etc.) on the coastal environment as well as to the monitoring of harmful algae in order to provide information to decision-makers. The current buoy benefits from 4 years of experience with the previous version deployed from 2004 to 2008. The current EOL model was deployed on April 15 2009 to collect multi-parameter profiles with near real-time data access, suitable for detecting any sporadic pulse change and for building long-term time series of physical and chemical parameters. This platform is a modular design integrating power supply (solar panels) and Internet connection. The EOL floats were designed with a special process (“roto molding”), from which the EOL buoy derives a strong and reliable resistance to waves and oscillations. The buoy is made of 4 stand-alone parts ensuring optimized buoyancy and able to resist to any collision. To prevent the development of biological activity on the sensors, the EOL platform uses a dedicated brush combined with chlorine application techniques (electrode) in order to automatically clean the sensors after each deployment. It offers both preventive and curative procedures prolonging the life of the sensors and guaranteeing high-quality data.

### Automated flow cytometry

An automated CytoSense flow cytometer (CytoBuoy, b.v., NL) was installed inside the EOL buoy. A computer connected to a WIFI antenna ensured the permanent remote control of the CytoSense. The WIFI connection was sufficiently powerful to download the data on a daily scale. The CytoSense was protected by a water-resistant cover preventing moisture penetration from large waves flowing inside the buoy. Samples were directly pumped from the EOL buoy side at 1 m depth and stored in a 500 cm^3^ intermediate container before analysis. The energy needed for the entire system was provided by 2 m^2^ solar panels throughout the experiment. The CytoSense is a flow cytometer specifically designed to analyse large phytoplanktonic cells (1–800 μm in width and a few mm in length) in relatively large volumes of water (several cm^3^ per sample). The seawater was pumped from the intermediate container with a calibrated (weighing method) peristaltic pump. The sheath fluid used to separate, align and drive the particles to the light source was continuously recycled using two sets of filters (porosity: 0.45 and 0.1 μm respectively). The sheath flow rate was 1.3 cm^3^ s^−1^. In the flow cell, each particle was intercepted by a laser beam (Coherent solid-state Sapphire, 488 nm, 15 mW) and the generated optical signals were recorded. The light scattered at 90° (sideward scatter, SWS) and fluorescence emissions were dispersed by a concave holographic grating and collected via a hybrid photomultiplier (HPMT). The forward scatter (FWS) signal was collected via a PIN photodiode. The red (FLR), orange (FLO) and yellow (FLY) fluorescences were collected in the wavelength ranges 734–668, 601–668, and 536–601 nm respectively. The stability of the optical unit and the flow rates were checked using Beckman Coulter Flowcheck™ fluorospheres (10 μm) before, during and after installation. Two distinct protocols were run sequentially, both triggered on the red fluorescence emission (induced by chlorophyll). The first one was set on the highest resolution in order to target autotrophic picoeukaryotes. FLR trigger level (threshold) was fixed at 7 mV, and sample flow rate at 4.5 mm^3^.s^−1^ for 4 min. The second was tuned for the analysis of nano- and microphytoplankton. FLR trigger was fixed at 10 mV, and sample flow rate at 9 mm^3^.s^−1^, for 10 min. The sampling schedule was programmed to sequentially run both analyses every 2 h.

The system failed to run from March 16, 10:00 am, to March 26, 12:00 am, due to a combination of technical and accessibility problems. Additional samples were therefore collected every 3 days during this period. They were fixed in 0.2% glutaraldehyde and stored at −80°C until analysis with a second CytoSense (CytoBuoy, b.v., NL) instrument provided by the PRECYM flow cytometry platform of MIO in order to record the abundances of the main phytoplankton clusters. Trigger levels and flow rates were set in order to obtain a population resolution similar to the buoy CytoSense settings.

CytoSense data were processed and analyzed with the CytoClus® software (CytoBuoy). Phytoplankton clusters were resolved using several two-dimensional cytograms of retrieved information (descriptors) from the 5 pulse shapes (FWS, SWS, FLO, FLR, FLY) obtained for each single cell, mainly the area under the curve and the maximum of the pulse shape signal.

### Conventional flow cytometry

Samples for ultraphytoplankton analysis were collected weekly at Point B station and immediately fixed with glutaraldehyde (1% final concentration), freeze-trapped in liquid nitrogen and stored at −80°C until analysis in the laboratory with conventional flow cytometry (Vaulot et al., 1989; Troussellier et al., 1995). Single-cell analysis was carried out using a Becton Dickinson FACSCalibur flow cytometer with a maximum flow rate of 1.08 mm^3^.s^−1^. The abundance of autotrophic prokaryotes and eukaryotes within the size class of pico-nano phytoplankton was assessed from unstained samples according to the method described by Marie et al. (1999).

### Nutrients analysis

For nutrients [NO^−^_3_; NO^−^_2_; PO^3−^_4_; Si(OH)_4_] analyses, 20 cm^3^ seawater samples were collected at 1 m every 2–5 days close to the CytoSense inlet on the EOL buoy, from January 31 to April 6. The samples were transferred to polyethylene flasks and directly frozen in the laboratory. Analyses were performed using a Technicon Autoanalyser® according to Tréguer and LeCorre (1975). Detection limits were 50, 20, 20, and 50 nM for NO^−^_3_, NO^−^_2_, PO^3−^_4_ and Si(OH)_4_ respectively.

### Chlorophyll analysis at point B

Total chlorophyll (chl) concentration was sampled at the Point B site and obtained by filtering 1 dm^3^ of seawater subsamples collected at 1 m onto 25-mm Whatman GF/F glass fiber filters and analysing them by fluorometry (Strickland and Parsons, 1972; SOMLIT protocol).

### Meteorological and hydrological data

Meteorological information was collected from a nearby Météo-France weather station (Nice airport station, 43.648°N, 7.208°E). Daily precipitation (mm), daily averaged wind speed (m.s^−1^) and daily solar radiance (J.cm^−2^) were the parameters selected to assess the influence of external events on marine water properties and phytoplankton community composition and dynamics.

Temperature (°C) and salinity were recorded every minute using a STPS sensor (nke instrumentation®) immersed at 2 m depth under the EOL buoy. This temperature/salinity data-set was compared with the SOMLIT Point B data-set collected weekly at 1 m (SBE25).

### Statistical analysis

Statistics were run under the R software (CRAN, http://cran.r-project.org/). Time series of phytoplankton abundances, nutrient concentrations, and hydrological and meteorological variables were smoothed using a loess method (library stats, function loess) followed by a predictive procedure (function predict; Cleveland and Devlin, 1988) in order to cover the periods of missing data and generate regular discrete time series with similar frequencies. The loess function corresponds to a local polynomial regression fitting. The weighted least squares local fitting uses neighborhood points with a tricubic weighting. Cross-correlation function (CCF) was computed between the environmental variables and the phytoplankton abundance's computed loess curves. Boxplot function was used to plot abundance variations over time.

## Results

### Water and meteorological variables

**Figure 2:**
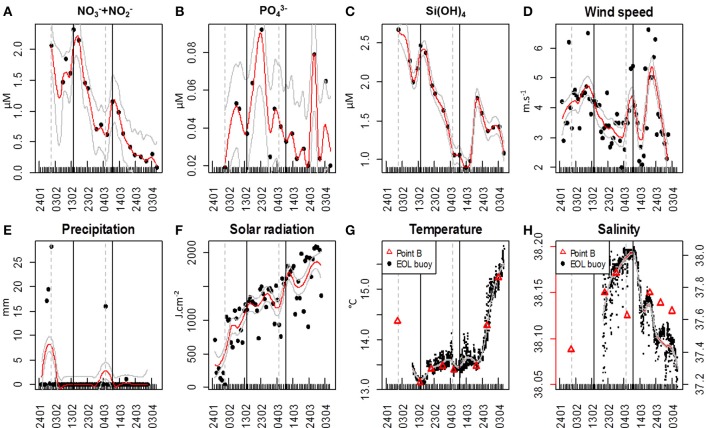
**Hydrological and meteorological characteristics in the vicinity of the EOL buoy**. Meteorological data were obtained from the Nice Meteo-France airport weather station (43.648°N, 7.208°E). Red lines represent the applied loess; continuous gray lines represent the loess standard error. The gray vertical dashed lines represent the two main precipitation events and the black continuous vertical lines represent the two main Nitrate + Nitrite pulses. **(A)** Nitrate + Nitrite (NO^−^_3_ + NO^−^_2_) concentrations (μM); **(B)** Phosphate (PO^3−^_4_) concentrations (μM); **(C)** Silicate (Si(OH)_4_) concentration (μM); **(D)** Daily averaged wind speed (m.s^−1^); **(E)** Daily precipitation (mm); **(F)** Daily global solar radiation (J.cm^−2^); **(G)** Water temperature at the EOL buoy (°C) and water temperature of the weekly sampling at Point B SOMLIT (red triangles); **(H)** Salinity at the EOL buoy and salinity of the weekly sampling at Point B SOMLIT (red triangles). Temperature and salinity below the EOL buoy were collected from February 9, 15:00, and from February 19, 21:00, respectively.

The sampling period was marked by two major NO^−^_3_ + NO^−^_2_ pulses (Figure 2A). The first NO^−^_3_ + NO^−^_2_ concentration pulse reached a maximum value of 2.31 μM on February 14; while the second pulse reached 1.15 μM on March 9. NO^−^_3_ + NO^−^_2_ concentration continuously decreased afterward until the end of the sampling period (Figure 2A). The first NO^−^_3_ + NO^−^_2_ peak was followed 9 days later by a sudden increase of PO^3−^_4_ concentration (0.09 μM on February 23, Figure 2B), whereas after the second NO^−^_3_ + NO^−^_2_ pulse, a large PO^3−^_4_ concentration increase (0.08 μM) was observed only 18 days later, on March 27 (Figure 2B). The first pulse of NO^−^_3_ + NO^−^_2_ coincided with a Si(OH)_4_ pulse (2.46 μM, Figure 2C). Si(OH)_4_ concentrations were maximal at the beginning of the sampling period (2.67 μM on January 24). A second pulse of Si(OH)_4_ was observed on March 20 (1.79 μM, Figure 2C), 11 days after the second NO^−^_3_ + NO^−^_2_ pulse. Four major wind events (wind speed >5 m.s^−1^) were recorded (Figure 2D). Two wind events took place before the first NO^−^_3_ + NO^−^_2_ pulse, on January 29 [6.2 m.s^−1^, northerly blowing (data not shown)] and on February 10 [6.5 m.s^−1^, south-easterly blowing (data not shown)]. A third coincided with the second NO^−^_3_ + NO^−^_2_ pulse and lasted for 2 days, on March 8 and 9 [5.3 and 5.4 m.s^−1^ respectively; blowing south-easterly (data not shown)]. The last wind event occurred on March 19 and was the most marked, with a wind speed of 6.6 m.s^−1^, south-easterly blowing (data not shown). It was followed by the second Si(OH)_4_ pulse (Figures 2C,D). Two major precipitation events were recorded. The first took place on January 31 with 28.2 mm of rainfall. The second resulted in 16.1 mm of rainfall on March 5 (Figure 2E). Solar radiation increased gradually throughout the sampling period, reaching a maximum daily average value of 2086 J.cm^−2^ (Figure 2F). Minimum water temperature was recorded by the *in situ* sensor on February 15 (13.1°C), whereas the maximum temperature was reached at the end of the sampling period (15.85°C on April 6; Figure 2G). Two dips in temperature occurred in coincidence with the two NO^−^_3_ + NO^−^_2_ pulses. Daily average temperature was 13.17 ± 0.03°C for three consecutive days [February 14, 15, and 16 (*n* = 72); 13.17 ± 0.03°C (*n* = 24); 13.16 ± 0.05°C (*n* = 24) and 13.16 ± 0.26°C (*n* = 24) respectively] before increasing again on February 17 (13.21 ± 0.08°C (*n* = 24)]. The second dip in temperature was in phase with the second NO^−^_3_ + NO^−^_2_ pulse and the third wind event (Figure 2G). The average water temperature between March 7 and March 10 was 13.44 ± 0.05°C (*n* = 96) while it was 13.53 ± 0.16°C (*n* = 24) on March 11. Point B average temperature was 13.8 ± 0.71°C (Figure 2G). Pearson correlation between Point B and EOL buoy temperature sensors was *r* = 0.97 (*n* = 7). Salinity values ranged from 32.92 to 38.04 (Figure 2H) with a mean of 37.51 ± 0.55 (*n* = 1369). There was a discrepancy between the Point B sensor and the EOL buoy sensor regarding salinity values. Point B average salinity was 38.14 ± 0.04 (Figure 2H). Pearson correlation between the two sensors was *r* = 0.88 (*n* = 5) once the Point B salinity value of March 6 was removed (extremely low value probably due to the local presence of freshwater after a spell of rain).

### Phytoplankton clusters

**Figure 3:**
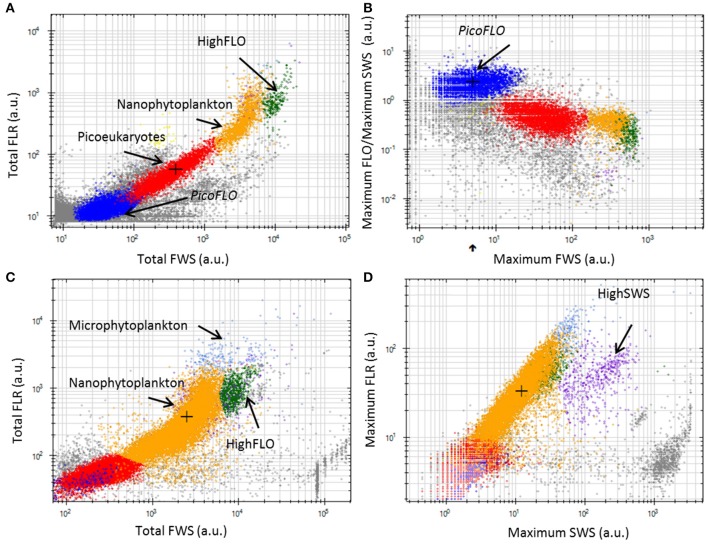
**Cytograms from the CytoClus software of samples analyzed with the CytoSense. (A)** Cytogram of Total red fluorescence [Total FLR (a.u.)] vs. Total forward scatter [Total FWS (a.u.)] with a red fluorescence trigger level of 7 mV allowing identification of PicoFLO, picoeukaryotes, nanophytoplankton, and HighFLO clusters. **(B)** Cytogram representing the ratio Maximum orange fluorescence/Maximum sideward scatter [Maximum FLO/Maximum SWS (a.u.)] vs. Maximum FWS with a FLR trigger level of 7 mV in which the PicoFLO cluster is distinguished. **(C)** Cytogram of Total FLR (a.u.) vs. Total FWS (a.u.) with a FLR trigger level of 10 mV permitting identification of nanophytoplankton, HighFLO, and microphytoplankton clusters. **(D)** Cytogram of Maximum FLR (a.u.) vs. Maximum SWS (a.u.) with a FLR trigger level of 10 mV in which the HighSWS cluster is made out. a.u., arbitrary unit.

A total of 6 phytoplankton clusters were distinguished over the 532 validated samples collected. The cell groups were discriminated on the basis of their optical properties, using two-dimensional data displays (cytograms, Figure 3). The smallest cells were observed when using the low FLR trigger level, as described in the Materials and Methods section (Figures 3A,B). The average volume analyzed with this protocol was 0.38 ± 0.13 cm^3^. The two main groups observed were labeled PicoFLO and picoeukaryotes (Figures 3A,B). The groups of cells with a higher FWS signature were observed using the high FLR trigger level and were labeled nanophytolankton, microphytolankton, HighSWS, and HighFLO (Figures 3C,D). The mean volume analyzed using the high FLR trigger level was 5.3 ± 1.15 cm^3^. Picoeukaryotes, nanophytoplankton, and microphytoplankton clusters were distinguished on the basis of their FLR and FWS signatures. PicoFLO and HighFLO clusters were discriminated on the basis of their FLO and FWS signatures, while the HighSWS group was identified by its high SWS signature.

**Figure 4:**
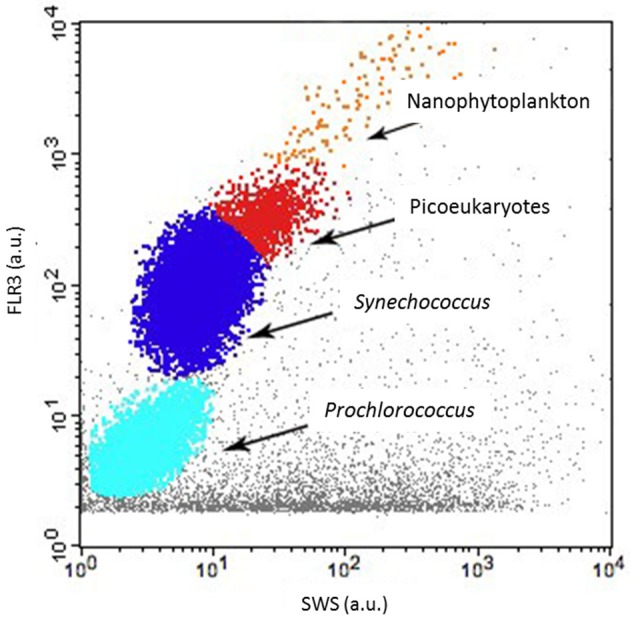
**Cytogram from the FACSCalibur flow cytometer with red fluorescence [FLR3 (a.u.)] vs. sideward scatter (SSC-H) showing the *Prochlorococcus* (not discussed in this paper), *Synechococcus*, picoeukaryotes and nanophytoplankton clusters**.

As regards conventional flow cytometry, four ultraphytoplankton groups were distinguished with the FACSCalibur flow cytometer over the study period on the basis of their optical signals (Li, 1994). *Synechococcus* (<1.5 μm) cells were resolved by their signature in a cytogram of red fluorescence (FL3, >620 nm) vs. orange fluorescence represented by phycoerythrin-containing pigment (FL2, 565–592 nm wavelength range). The *Prochlorococcus* (<1 μm) cluster exhibits smaller scatter intensities than *Synechococcus*, a lower red fluorescence signal and no orange fluorescence signal. Data from this latter group are not included in this paper. Picoeukaryotes (<2 μm) and nanophytoplankton (2–10 μm) cells were resolved in red fluorescence vs. side scatter plots (Figure 4).

### Abundance trends and pulses

**Figure 5:**
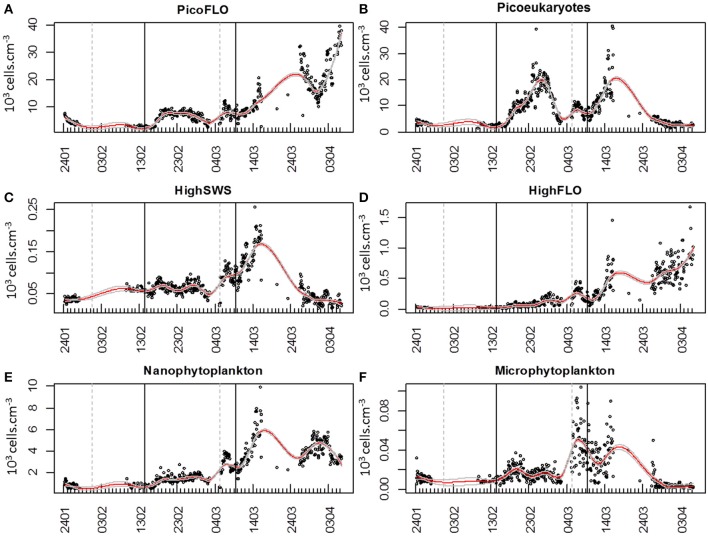
**Dynamics of cell abundances as determined with CytoBuoy's instrument for each resolved cluster**. The gray vertical dashed lines materialize the two main precipitation events (Figure 2E) and the black vertical lines the two main NO^−^_3_ + NO^−^_2_ pulses (Figure 2A). Continuous red lines represent the applied loess to the time series, with its standard error (gray continuous lines). **(A)** Abundance of PicoFLO cells (10^3^ cells.cm^−3^). **(B)** Abundance of picoeukaryote cells (10^3^ cells.cm^−3^). **(C)** Abundance of HighSWS cells (10^3^ cells.cm^−3^). **(D)** Abundance of HighFLO cells (10^3^ cells.cm^−3^). **(E)** Abundance of nanophytoplankton cells (10^3^ cells.cm^−3^). **(F)** Abundance of microphytoplankton cells (10^3^ cells.cm^−3^).

**Figure 6:**
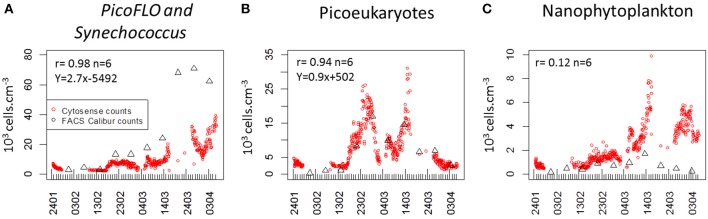
**Comparison of abundances determined with the FACSCalibur flow cytometer (black triangles) and the CytoSense flow cytometer (red dots)**. Pearson rank correlation [(r) between the two counts is given in each panel. **(A)** PicoFLO cells (10^3^ cells.cm^−3^). **(B)** Picoeukaryote cells (10^3^ cells.cm^−3^). **(C)** nanophytoplankton cells (10^3^ cells.cm^−3^).

PicoFLO abundance analyzed with the CytoSense flow cytometer varied between 2320 and 39,400 cell.cm^−3^ (mean: 9583 ± 7401 cells.cm^−3^; Figure 5A). *Synechococcus* abundance analyzed with the FACSCalibur flow cytometer ranged from 3223 to 52,810 cell.cm^−3^ (mean: 28,183 ± 27,688 cells.cm^−3^; Figure 6A). CytoSense counts were much lower than counts from the FACSCalibur flow cytometer due to the specific CytoSense configuration used during this experiment, in which the photomultiplier tubes were not sensitive enough to detect these dimly fluorescent cells. The correlation between the two instruments regarding these abundance measurements was significant (*r* = 0.98, *n* = 6, Pearson, samples from the Cytosense collected within 2 h from the FACSCalibur sampling). Picoeukaryotes abundance varied between 1401 and 40,280 cells.cm^−3^ (mean: 7875 ± 6508 cells.cm^−3^, Figure 5B) with the CytoSense instrument, while they varied between 426 and 17,000 cells.cm^−3^ (mean: 6876 ± 5743 cells.cm^−3^, Figure 6B) with the FACSCalibur flow cytometer. In this case, abundances were significantly correlated (*r* = 0.94, *n* = 6, Pearson). HighSWS abundance was only assessed with the CytoSense instrument, based on their high SWS signature (Figure 3C). Their abundance varied between 15.11 and 256 cells.cm^−3^ (mean: 65 ± 37 cells.cm^−3^, Figure 5C). HighFLO cells were detected with the CytoSense instrument only. Their abundance varied between 6 and 1676 cells.cm^−3^ (mean: 226 ± 275 cells.cm^−3^, Figure 5D). Nanophytoplankton abundance as recorded by the CytoSense instrument ranged from 495 to 9888 cells.cm^−3^ (mean: 2260 ± 1631 cells.cm^−3^, Figure 5E), whereas with the FACSCalibur flow cytometer it varied between 190 and 1728 cells.cm^−3^ (mean: 703 ± 443 cells.cm^−3^, Figure 6C). Correlation between the CytoSense and the FACSCalibur flow cytometer regarding nanophytoplankton counts was not significant although values followed similar trends (Figures 5E, 6C). The microphytoplankton cluster was only observed with the CytoSense instrument, with cell abundances between 0 and 103 cells.cm^−3^ (mean: 17 ± 16 cells.cm^−3^, Figure 5F).

**Figure 7:**
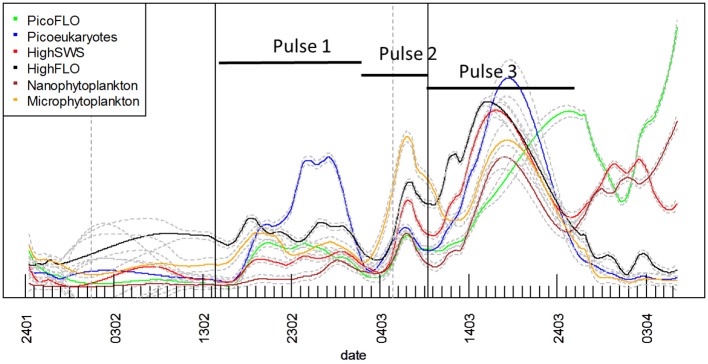
**Superimposition of each cluster's predicted loess results derived from the raw abundance time series**. All three different abundance pulses involved all phytoplankton clusters. The abundances of the different clusters are not represented to scale on the Y-axis for the sake of clarity (see Figure 5 for each cluster's abundance variations). The gray vertical dashed lines materialize the two main precipitation events (Figure 2E) and the black vertical lines the two main NO^−^_3_ + NO^−^_2_ pulses (Figure 2A). Continuous lines represent the loess applied to the time series, with its standard error (gray dashed lines).

The time course of cell abundances within each cluster optically resolved in the CytoSense data-set was smoothed with the loess procedure and missing values were predicted at hourly intervals. The smoothed curves are superimposed (Figure 7) to highlight possible succession patterns induced by environmental perturbations during the experiment.

The six clusters investigated behaved similarly throughout the sampling period, evidencing three main abundance pulses (Figure 7). The slope of the loess function at the hourly interval was calculated and the balance between negative and positive slope gave the time when abundances increased. The loess procedure may affect the precision of the exact time of the beginning of the abundance pulse, as the estimated standard error shows (dashed gray curve, Figure 7). The first abundance pulse (Pulse 1, Figure 7) occurred starting February 16 for PicoFLO, picoeukaryotes, nanophytoplankton, HighSWS and HighFLO clusters. Microphytoplankton abundance had risen the day before, starting February 15 (Figure 7). The second cell-abundance pulse (Pulse 2, Figure 7) was initiated by the picoeukaryotes and microphytoplankton clusters on March 2, followed by all the remaining clusters after March 3. The third cell-abundance pulse (Pulse 3, Figure 7) took place starting March 9 for the picoeukaryotes and the HighSWS clusters, coinciding with the second NO^−^_3_ + NO^−^_2_ pulse and the second south-easterly wind event (Figures 2A,D). The nanophytoplankton, HighFLO and PicoFLO clusters started to increase on March 10, and microphytoplankton on March 12 (Figure 7).

During this third cell-abundance pulse, we identified the following 4-step pattern of succession: 1- picoeukaryotes and HighSWS, 2- nanophytoplankton, 3- HighFLO, and PicoFLO, 4- microphytoplankton (Figure 7). The PicoFLO abundance pulse started later than the other groups and peaked on March 26 (36,121 cells.cm^−3^) as well as on the last day of sampling (April 6, 37,975 cells.cm^−3^). *Synechococcus* abundance determined with the FACSCalibur flow cytometer showed a similar trend but the sampling strategy could not provide evidence for the last pulse (Figure 6A). This limitation also concerns abundances of picoeukaryotes and nanophytoplankton determined with the FACSCalibur (Figures 6B,C). Although the CytoSense did not run from March 16 to March 26, the nanophytoplankton and HighFLO clusters were the only ones to see their cell count rise after March 26 (Figure 7), 6 days after the latest observed wind event >5 m.s^−1^ (Figure 2D).

### Cross-correlation between abundance trends and environmental variables

A cross-correlation between environmental variables and clusters' abundance pulses was computed to determine the lag of highest correlation between each selected variable (Table 1). All clusters were taken into account, with the exception of the PicoFLO cluster after we found evidence of uncertainty regarding its abundance counts (Figure 6A). Similarly, salinity data collected at the EOL buoy were not used due to the discrepancy observed between its sensor and the Point B sensor (Figure 2H). Cross-correlations integrating abundance Pulse 2 and Pulse 3 (Figure 7) were run 1 day before in order to integrate as much as possible the preceding environmental conditions. During abundance Pulse 1 (Figure 7), cross-correlation between the clusters' abundance and NO^−^_3_ + NO^−^_2_, PO^3−^_4_, wind speed and solar radiation respectively were all significant (Table 1). Cross-correlation between PO^3−^_4_ and solar radiation was the highest observed and occurred with a lag of less than 5.2 days (Table 1). During abundance Pulse 2, PO^3−^_4_, Si(OH)_4_, precipitation, and temperature were correlated with all the clusters' abundance increase, with lags not exceeding 5.2 days (Table 1). The drop in Si(OH)_4_ concentration is correlated with the end of the abundance pulse, 5 days later, when the clusters' abundance decreased (Figures 2C, 7). The highest cross-correlation was observed between clusters' abundance and precipitation, with lags of under 2 days (Table 1). During abundance Pulse 3 (Figure 7), all clusters were correlated with NO^−^_3_ + NO^−^_2_ and four of them with precipitation (Table 1), with lags between 4.6 and 10.7 days.

**Table 1:** **Cross-correlation between the environmental variables and the abundance loess curves (Figures 2, 5) during the three main abundance pulses described in Figure 7**.

Period DD/MM/YY	Cluster abundance/variable Loess curve	NO^−^_3_ + NO^−^_2_	PO^3−^_4_	Si(OH)_4_	Precipitation	Wind speed	Solar radiation	Temperature
31/01/2012–01/03/2012 *n* = 901	**Picoeukaryotes**	*r* = 0.52 lag = 9.2	*r* = 0.82 lag = 3.1	*r* = 0.47 lag = 21.5	*r* = 0.73 lag = 25.6	*r* = 0.52 lag = 15.8	*r* = 0.65 lag = 0.7	
	**HighSWS**	*r* = 0.29 lag = 9.2	*r* = 0.46 lag = 0			*r* = 0.30 lag = 15.9	*r* = 0.89 lag = 0	
	**HighFLO**	*r* = 0.50 lag = 11.1	*r* = 0.80 lag = 5.2		*r* = 0.74 lag = 27.6	*r* = 0.53 lag = 17.4	*r* = 0.70 lag = 1.7	*r* = 0.90 lag = 0
	**Nano**	*r* = 0.44 lag = 9.4	*r* = 0.70 lag = 2.5	*r* = 0.38 lag = 22.3	*r* = 0.59 lag = 26.3	*r* = 0.50 lag = 16.6	*r* = 0.82 lag = 1.4	
	**Micro**	*r* = 0.32 lag = 4.4	*r* = 0.67 lag = 0	*r* = 0.36 lag = 15.7	*r* = 0.50 lag = 20.5	*r* = 0.41 lag = 15.8	*r* = 0.74 lag = 2.6	
01/03/2012–08/03/2012 *n* = 191	**Picoeukaryotes**		*r* = 0.34 lag = 3.2	*r* = 0.38 lag= 4.5	*r* = 0.75 lag = 1.4	*r* = 0.57 lag = 0.4		*r* = 0.30 lag = 4.0
	**HighSWS**		*r* = 0.45 lag = 3.8	*r* = 0.48 lag = 5.2	*r* = 0.73 lag = 1.4	*r* = 0.98 lag = 0		*r* = 0.43 lag= 4.4
	**HighFLO**		*r* = 0.45 lag = 3.1	*r* = 0.49 lag = 4.4	*r* = 0.80 lag = 0.9			*r* = 0.42 lag = 3.8
	**Nano**		*r* = 0.44 lag = 3.6	*r* = 0.47 lag = 4.9	*r* = 0.75 lag = 1.2	*r* = 0.94 lag = 0		*r* = 0.42 lag = 4.2
	**Micro**		*r* = 0.43 lag = 3.5	*r* = 0.46 lag = 4.7	*r* = 0.74 lag = 1			*r* = 0.42 lag = 4.1
08/03/2012–06/04/2012 *n* = 764	**Picoeukaryotes**	*r* = 0.59 lag = 6.7	*r* = 0.3 lag = 7.12		*r* = 0.39 lag = 10.7		*r* = 0.31 lag = 6.3	
	**HighSWS**	*r* = 0.69 lag = 4.6	*r* = 0.35 lag = 6.2		*r* = 0.38 lag = 9.8	*r* = 0.43 lag = 9.5	*r* = 0.41 lag = 5.3	
	**HighFLO**	*r* = 0.45 lag = 7.2						*r* = 0.37 lag = 0
	**Nano**	*r* = 0.57 lag = 5.8			*r* = 0.32 lag = 9.8			
	**Micro**	*r* = 0.51 lag = 6.0	*r* = 0.34 lag = 6.8		*r* = 0.41 lag = 10.6	*r* = 0.44 lag = 9.7	*r* = 0.47 lag = 5.9	*r* = 0.30 lag = 14.3

In Pulse 2 and 3 (Figure 7), the cross-correlation period was started 1 day before the pulse's onset in order to better assess the influence of the environmental variables on each cluster's abundance. Time lags are indicated in days.

### Daily cycles

**Figure 8:**
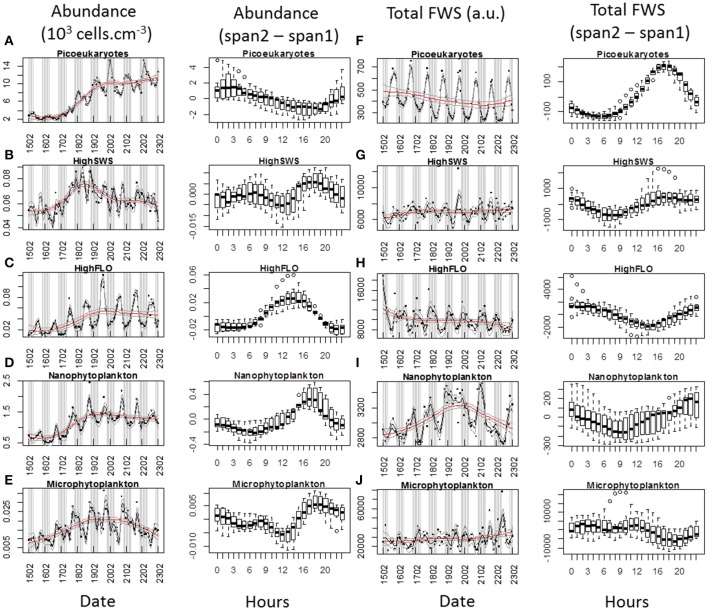
**Periodic variations of abundances and average Total FWS signals per cell for each cluster resolved with the CytoSense (except PicoFLO) during the first marked abundance pulse (February 15–22)**. The corresponding hourly box plots are displayed in parallel. Note the differences between a low span (gray line: span2) and a high span (red line: span1) loess procedure for both variables. Abundance (10^3^ cells.cm^−3^) and difference between span1 and span2: **(A)** picoeukaryotes. **(B)** HighSWS. **(C)** HighFLO. **(D)** nanophytoplankton. **(E)** microphytoplankton. Average cell Total FWS (a.u.) and difference between span1 and span2: **(F)** picoeukaryotes. **(G)** HighSWS. **(H)** HighFLO. **(I)** nanophytoplankton. **(J)** microphytoplankton. a.u., arbitrary unit.

**Figure 9:**
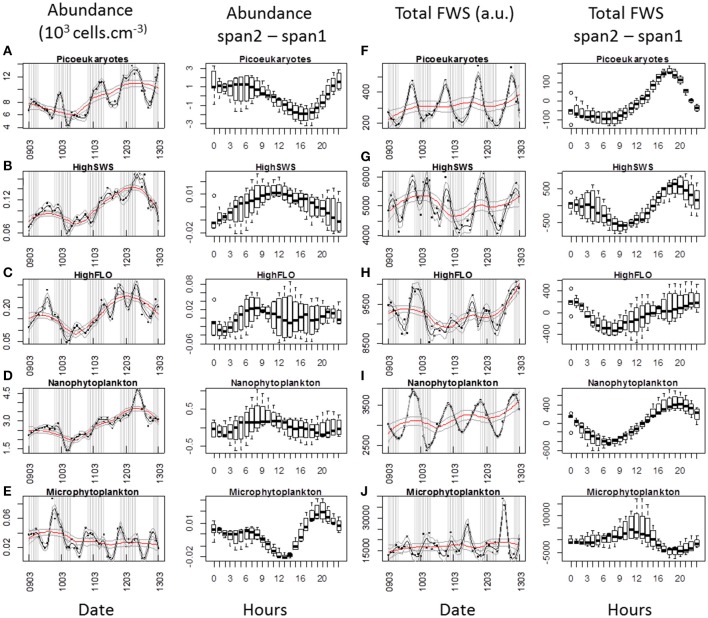
**Periodic variations of abundances and average Total FWS signals per cell for each cluster resolved with the CytoSense (except PicoFLO) during the third marked abundance pulse (March 9–13)**. The corresponding hourly box plots are displayed in parallel. Note the differences between a low span (gray line: span2) and a high span (red line: span1) loess procedure for both variables. Abundances (10^3^ cells.cm^−3^) and difference between span1 and span2: **(A)** picoeukaryotes. **(B)** HighSWS. **(C)** HighFLO. **(D)** nanophytoplankton. **(E)** microphytoplankton. Average cell Total FWS (a.u.) and difference between span1 and span2: **(F)** picoeukaryotes. **(G)** HighSWS. **(H)** HighFLO. **(I)** nanophytoplankton. **(J)** microphytoplankton. a.u., arbitrary unit.

When analysing phytoplankton at the hourly scale by flow cytometry, it is possible to detect *in situ* diel cycles. Applying different loess spans is a way to separate the daily periodic information from the longer-term trend (Figures 8, 9). For each cluster, the daily variations in cell abundance and cell average FWS were plotted throughout abundance pulses for which the data-sets were almost complete, i.e. the first and third pulses [February 15–23 (Figure 8) and March 9–13 (Figure 9) respectively]. The diel variations of PicoFLO abundance are not reported here because of the large discrepancy between the values generated by the CytoSense and the FACSCalibur instruments. The CytoSense's under-sampling, although it affected the absolute values only and not the abundance trend, could have seriously distorted the average FWS and FLR intensities of this cluster.

The median values obtained from the boxplots show that picoeukaryotes abundance increased between 19:00 and 2:00 (Figure 8A) during the first pulse, vs. between 17:00 and 22:00 (Figure 9A) during the third pulse. Both pulses saw the FWS intensities peak at 18:00 (Figures 8F,F). HighSWS abundance increased between 13:00 and 18:00 (Figure 8B) vs. between 23:00 and 10:00 (Figure 9B). FWS values reached their maximum at 18:00 (Figure 8G) vs. 17:00 (Figure 9G). HighFLO cell abundance increased between 5:00 and 13:00 (Figure 8C) vs. between 2:00 and 8:00 (Figure 9C). Maximum FWS values were recorded at 0:00 (Figure 8H) vs. 22:00 (Figure 9H). Nanophytoplankton abundance increased between 8:00 and 17:00 (Figure 8D) vs. between 2:00 and 9:00 (Figure 9D). Nanophytoplankton reached maximum FWS values at 22:00 (Figure 8I) vs. 20:00 (Figure 9I). Microphytoplankton abundance increased between 12:00 and 18:00 (Figure 8E) vs. between 13:00 and 20:00 (Figure 9E). FWS intensities peaked at 11:00 (Figure 8J) for the first event vs. 2:00 and 11:00 (Figure 9J) for the other. Most of the daily abundance increases recorded started later in the day in the first abundance pulse event (Figure 8) than in the other pulse event illustrated (Figure 9). The only exceptions are the HighSWS and microphytoplankton clusters, for which diel abundance increased approximately 12 and 1 h earlier, respectively, in the first abundance pulse than in the third.

## Discussion

The data obtained by combining two innovative technologies (full auto-powered buoy and automated remote-controlled flow cytometry) delivered results and information that are not only never provided by conventional sampling procedures but also beyond their scope. Hourly-scale observation of the phytoplankton community structure was previously established *in situ* from a harbor (Thyssen et al., 2008b; Campbell et al., 2010), from a submersible instrument close to a harbor (Sosik et al., 2003; Olson and Sosik, 2007) and from “ships of opportunity” (Thyssen et al., 2009; Ribalet et al., 2010). But the experiment described in this paper is the first one to have been conducted on a self-powered system moored away (approximately 1.7 km) from a Wi-Fi hotspot through which the equipment was controlled. Throughout this two-month study, it was possible to observe the hourly and weekly changes in the phytoplankton community structure at a fixed point. The collected data-set gave us the opportunity to pinpoint the onset of the spring phytoplankton bloom, determine the associated environmental conditions, and identify succession patterns of the different phytoplankton functional groups involved. However, in order to fully integrate the role of phytoplankton in sustaining the marine environment, trophic status and biogeochemical processes (Mével et al., 2008; Riser and Johnson, 2008; Schneider et al., 2008; Finkel et al., 2010), combining fixed point sampling and spatial coverage is a key issue.

### Cluster selection and identification from flow cytometry

*Synechococcus* and larger phycoerythrin-containing cryptophytes are identified by their SWS, FLR and FLO signatures (Olson et al., 1988; Veldhuis and Kraay, 2000; Marie et al., 2010). Picoeukaryotes and nanophytoplankton clusters are mostly identified on the basis of their FLR and FWS/SWS signatures. Similarly, cluster identification using pulse shape CytoSense instruments is based on recorded pulse optical properties. Optical pulse shapes recording improves the cells' characterization compared with the peak of the optical signal routinely recorded from conventional flow cytometers. In this study, no sorting followed by microscopic analyses was run to validate the CytoSense's attributions to the various functional clusters. Picoeukaryotes, nanophytoplankton, and microphytoplankton are defined on the basis of FWS (related to size) and FLR (related to chlorophyll content) signatures. This group may include chain-forming cells, in which case the unit “cells.cm^−3^” has to be considered with attention, but in our study, chain-forming cells were not significantly counted. The optical signatures of PicoFLO, HighFLO, and HighSWS are similar to those of functional groups identified through conventional flow cytometry, i.e., *Synechococcus* (Olson et al., 2003), Cryptophytes (Marie et al., 2010), and Coccolithophores (Veldhuis and Kraay, 2000; von Dassow et al., 2012), although flow cytometry can only detect Coccolithophore cells that bear coccoliths due to their particular light scatter properties. The version of the CytoSense instrument used in this study (low sensitivity of photomultiplier tubes) does not differentiate *Prochlorococcus* cells from the noise and cannot provide reliable *Synechococcus* countings (Figure 6A). *Synechococcus* could have been adequately counted if FLO or SWS had been used for triggering instead of FLR. On the other hand, conventional flow cytometry cannot span the entire size range of nanophytoplankton (2–20 μm, Figure 6C) due to its limit regarding the volume analyzed (close to 400 mm^3^). In this study, the two instruments used in conjunction compensate each other's weaknesses and fill in each other's gaps.

### Phytoplankton abundance dynamics and environmental changes

In our study, changes in NO^−^_3_ + NO^−^_2_ concentrations, in wind speed and direction as well as in rainfall revealed the potential role of these environmental variables in phytoplankton community dynamics in this particular area (Figures 2, 5, 7, Table 1), even though wind speed never actually reached what is defined as a strong wind event (>10m.s^−1^; Warembourg, 2005) in the course of our two-month experiment. Concentrations of NO^−^_3_ + NO^−^_2_ were consistent with values previously observed during this period in the same area (Vandromme et al., 2011). The three main increases in phytoplankton abundance involving the clusters resolved by the CytoSense closely followed environmental modifications, at least as far as we could interpret them within the limits of the environmental variables sampled.

The comparisons between the EOL buoy sampling site and the Point B SOMLIT took in consideration the fact that both sites are distant of 355 m. The Bay of Villefranche-sur-Mer was shown to be homogeneous from July to March (Nival and Corre, 1976). The two sampling sites have shown similar temperature values and picoeukaryotes counts, suggesting that those clusters are homogeneously distributed between the two sample points. Due to technical differences between the two instruments, only picoeukaryotes analyses could be used to validate the CytoSense counting. The low and negative Pearson correlations for nanophytoplankton comparison and the slope difference for the PicoFLO/*Synechococcus* comparison clearly show that the counts were dissimilar, but the successive increase and decrease in abundance recorded around March 14 are visible in both data-sets. The FACSCalibur instrument is more specialized on Nanophytoplankton under 10 μm, while the CytoSense instrument resolves cells up to 800 μm (Figures 3, 4).

The first increase in phytoplankton abundance occurred after a moderate wind event (February 10; Figure 2D) that may have been at the origin of the NO^−^_3_ + NO^−^_2_ pulse (Figure 2A), of the Si(OH)_4_ pulse (Figure 2C) 4 days later, and of a decrease in temperature (Figure 2G). Easterly winds are common in spring (Warembourg, 2005), may generate open water inclusions (Nival et al., 1975) and could be related to surges in nutrient content (Warembourg, 2005). The observed nutrient pulse triggered an increase in abundances in all described groups (Table 1), and more particularly in picoeukaryotes (Figures 5B, 7), with an up to 40-fold increase within 10 days followed by a collapse to initial values within 5 days. This first increase in abundance, mainly involving pico- and nanophytoplankton, is commonly observed in the area before the spring bloom (Gomez and Gorsky, 2003). The fact that the largest cells did not react as much as during the second and third pulses can be reasonably explained by the fast nutrient depletion [especially NO^−^_3_ + NO^−^_2_ and Si(OH)_4_; Figures 2A,C] and by the subsidence in solar radiation (Figure 2F). On March 2, phytoplankton concentrations had dropped within 4 days to close to their initial January values, in parallel with the dissipation of the first pulse of NO^−^_3_ + NO^−^_2_ (Figure 2A).

**Figure 10:**
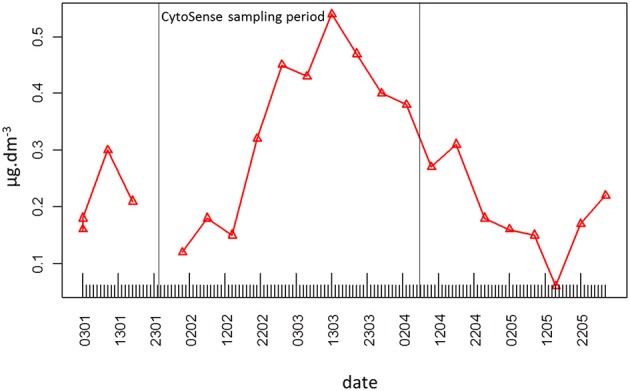
**Weekly surface water chlorophyll concentration (μg.dm^−3^) at Point B throughout the experiment**.

The second pulse in phytoplankton abundance started in relation with the precipitation event of March 5 (Figures 2E, 5, 7, Table 1). This pulse also involved all the clusters resolved with the CytoSense, but the increase in picoeukaryotes abundance was much weaker than the previous one (picoeukaryotes abundance only increased 3-fold; Figures 5B, 7). The other clusters reached higher abundances compared with the first pulse, especially microphytoplankton cells, which reached their maximum abundance values (Figure 7). This pulse was not evidenced by the FACSCalibur data-set (Figure 6). A slight surge in NO^−^_3_ + NO^−^_2_, PO^3−^_4_ and Si(OH)_4_ concentrations was recorded on March 2 (Figures 2A–C). Clusters' abundance reacted within 3 days to this PO^3−^_4_ concentration rise (Figure 2B, Table 1). The precipitation event occurred later, suggesting that the second abundance pulse was driven by the nutrients (Table 1). This short abundance pulse was probably terminated by the wind event observed on March 9 (Figure 2D), which coincided with a NO^−^_3_ + NO^−^_2_ pulse (Figure 2A) and a temperature decrease (Figure 2G). Conversely, this wind event would have induced the third phytoplankton abundance pulse (after March 9), together with the elevation in surface water temperature (Figure 2G) and augmentation of solar radiance (Figure 2F). High correlations with a lag of 4.6–7.2 days were observed between NO^−^_3_ + NO_2_ and all phytoplankton clusters (Table 1). The lag recorded after the precipitation event was longer (i.e. 9.8–10.7 days), suggesting that this event (certainly in association with wind) was conducive to the NO^−^_3_ + NO_2_ concentration surge. This third pulse was the longest and the most intense we observed during the sampling period, and it could be related to the spring phytoplankton bloom usually observed in this area (Gomez and Gorsky, 2003; Vandromme et al., 2011). This bloom also corresponded to the maximum chlorophyll concentration recorded at Point B during the weekly SOMLIT survey (Figure 10). The last wind event observed, on March 19, blowing at a speed >6 m.s^−1^, could have triggered the sudden cell abundance collapse recorded in nearly all the phytoplankton clusters. This phenomenon was also observed with easterly wind events (Warembourg, 2005), although these were not particularly marked.

The abundance pulses recorded and described in this study using high-frequency sampling shed new light on the way phytoplankton is blooming after the winter mixing period, compared with conventional sampling strategies. The deeper understanding of the successive meteorological and nutrient events permitted by the hourly sampling strategy is the only way to quantify the role of each environmental factor in determining biomass production. The photosynthetic picoeukaryotes group is a quick-reacting group when one considers its sharp increases in abundance. It is mainly abundant in oligotrophic areas and promptly responds to the mesotrophic/oligotrophic transition (Denis et al., 2003), in relation with nutrient pool availability (Veldhuis et al., 2005). In the course of this study, two major increases in picoeukaryotes abundance were recorded (the first and third abundance pulses). Both led to a more than 10- to 20-fold abundance increase, but the first one was not followed by any sizeable proliferation of larger cells, and was described as a first pico-nanophytoplankton bloom (Gomez and Gorsky, 2003). The second pulse of picoeukaryotes abundance opened the way to the described and expected spring phytoplankton bloom, in which larger cells reached high abundances as well (Gomez and Gorsky, 2003). Picoeukaryotes certainly play a role in the onset of the phytoplankton spring bloom and in the succession pattern (*via* nutrient release through excretion, viral lysis and/or grazing), but it also appears that external environmental events such as wind events (Lacroix and Nival, 1998) and water mixing (Bustillos-Guzman et al., 1995) combined with sufficient light availability contribute to controlling the further development of the larger species, as expected during the diatom/large cell phytoplankton spring bloom.

### Phytoplankton diel cycles

We succeeded in shedding some light on the cell cycle, which drives the growth rate, by considering diel variations of abundance together with FLR and FWS intensities measured at the single-cell level (Chisholm and Brand, 1981; DuRand and Olson, 1996; Vaulot and Marie, 1999; Sosik et al., 2003; Thyssen et al., 2008b). Calculation of *in situ* growth rates can be used (Sosik et al., 2003; Dugenne et al., this issue) to better estimate the system's losses and phytoplankton biomass production (Andre et al., 1999). In this paper, we interpret the diel variations in terms of abundance and FWS periodicity only. Maximum FWS values are related to the G2 phase of the cell cycle, before the cell division that leads to an increase in abundance. Diel abundance increases could be related to the cell cycle, but the expected increase is usually not seen *in situ*, in part because of losses such as grazing, advection/convection and viral lysis. The two main pulses in phytoplankton abundance (starting February 15 and March 9, Figures 8, 9 respectively) were selected because fast growth rates are expected during these periods. Unsurprisingly, the maximum abundance values recorded corresponded to the FWS minimum values (Figures 8, 9), in agreement with the fact that mitosis is followed by an increase in abundance (Jacquet et al., 1998; Vaulot and Marie, 1999). The combination of both cell cycle proxies was remarkably well defined during this particular period characterized by a well-mixed water column (Gomez and Gorsky, 2003). Both clearly exhibited abundance and FWS diel periodicities, but with some differences regarding the time at which peak values were reached. This suggests a change such as a delayed cell cycle, or a modification of the species composition of the clusters. The abundance increase started later in February than in March for picoeukaryotes, nanophytoplankton and HighFLO, as well as their respective FWS maxima (except for picoeukaryotes). This means cells begin dividing at a later hour when the sunset takes place earlier.

Picoeukaryotes abundance increased during the night while FWS increased until 17:00–18:00, indicating cell growth during the day and cell division after sunset (Jacquet et al., 2001, 2002; Thyssen et al., 2008b). Diel variations in abundance were well pronounced, especially in March (Figure 8A), which was not the case during the July study by Jacquet et al. (1998). The cell abundance periodicity occurring during the recorded abundance pulses could be linked to nutrient availability, better light conditions as well as to a homogenized water column, in contrast with the stratified period of July (Gomez and Gorsky, 2003). The abundance periodicity of HighSWS cells was rather subdued during the first phytoplankton abundance pulse in February, whereas in March both abundance and FWS periodicities were well marked (Figures 8B,G, 9B,G). The time interval between the FWS abatement after its maximum value recorded at 16:00 and the increase in abundance that occurred at midnight could be due to a delay within the cell cycle. The HighFLO cluster exhibited a strongly marked cycle during the few days selected in February (Figures 8C,H), with division in the early hours (around 3:00, Figure 8H) and an increase in cell abundance in opposite phase. A similar but less pronounced pattern of abundance and FWS was recorded during the period selected in March (Figures 9C,H). Night-time division of HighFLO cells with a clear diel periodicity in cell abundance has previously been reported by Jacquet et al. (2002) in winter in the modified Atlantic waters of the Alboran Basin. In our study, HighFLO cells were sometimes difficult to single out on the basis of their orange fluorescence, possibly because of low phycoerythrin cell content. However, a cluster was clearly distinguished on the FLR vs. FWS cytogram (Figure 3A), with cells characterized by a weak intensity orange fluorescence, distinctly separate from the nanophytoplankton cluster.

In February, the nanophytoplankton cluster exhibited a clear periodicity regarding cell abundance, but none in the case of FWS. The reverse was true in March, when abundance periodicity was more subdued than FWS periodicity (Figures 8D,I, 9D,I, respectively). This could result from predators (ciliates) grazing on the various species that make up the nanophytoplankton cluster (Rassoulzadegan et al., 1988). Because of the small volume analyzed with conventional flow cytometry (typically less than half a cm^3^), nanophytoplankton abundance is often underestimated and nanophytoplankton cycles are consequently poorly documented. This constraint is alleviated when using a dedicated automated flow cytometer such as the one we employed in this study, capable of analysing up to 5 cm^3^, i.e., nearly ten times more than conventional flow cytometers. Microphytoplankton maximum FWS intensities were observed around midday in both selected events (11:00, Figures 8J, 9J), suggesting that division took place during the day-time. This was also documented in some diatom species (Chisholm and Brand, 1981; Vaulot et al., 1986), in which cell division could be synchronized, depending on light and nutrient limitations (Mocquet et al., 2013). During the selected February event, the N/Si ratio kept above 0.95 until February 17, while during the selected March event, the N/Si ratio exceeded 1.1 (data not shown), suggesting no nutrient limitation. Furthermore, the abundance and FWS periodicities on March 11, 12, and 13 were characterized by increases taking place both in the morning and at dusk. This recalls the two daily optimal division timings seen in diatoms (Mocquet et al., 2013).

Although grazing was not investigated in this study, it is well established that it controls organic matter production in the area (Rassoulzadegan and Sheldon, 1986). When considering the recorded cell abundance periodicities and the occasional population collapses, it is reasonable to invoke top-down control by various grazing species on selected size classes (Bernard and Rassoulzadegan, 1993), or even vertical migration of either phytoplankton cells or microzooplankton, as well as water-column mixing. The pico- and nanophytoplankton abundances increased very fast between February 17 and February 20 (Figures 8A,D). These sudden increases in abundance, superimposed on abundance periodicities, may result from a combination of high division rate and low grazer abundance.

## Conclusion

Abundances and optical properties of phytoplankton communities were monitored *in situ* at the single cell level, at the hourly scale over nearly 2 months in the northwestern Mediterranean Sea using a totally autonomous and remotely controlled facility. To our knowledge, this is an unprecedented achievement. The flow cytometer used in this study is specially designed for phytoplankton analysis (combining wide flow cell and large volume analysis) and offers an appropriate means to undertake in near real time the *in situ* study of phytoplankton functional types and their dynamics. It provides an hour-by-hour access to phytoplankton community structure and composition exposed to environmental changes. With such high-frequency readings, taking into account the short-term variability of phytoplankton is now possible. This is of great importance to better understand the role of the primary producers, since we know that cells alter their cell kinetics on a very short time scale. In coastal areas, the conditions that lead to the onset of the phytoplankton spring bloom arise from several complex factors, to which the phytoplankton community structure responds differently depending on its composition. The concept of response functional groups stems from this behavior (Thyssen et al., 2008b). We here highlighted the influence of rain, wind events and nutrient pools in triggering different pico-nanophytoplankton blooms before the onset of the actual microphytoplankton bloom. The number and amplitude of abundance pulses from the various phytoplankton size classes and functional groups changed on the scale of the day over this 2 months experiment. As stated by Lomas et al. (2009), not taking into account fast biomass pulses of pico-nanophytoplankton could drastically alter the annual-scale estimations of biogeochemical fluxes and budgets. Observing phytoplankton at the single cell level and *in situ* is a fantastic opportunity to gain a deeper understanding of its behavior in response to its environment. The next generation of automated instruments that will allow *in situ* staining, incubation and analysis of marine samples with fluorescent dyes (such as nucleic acids dyes, physiological probes, etc.) will further our comprehension of population dynamics and biogeochemical impacts on larger scales encompassing not only phytoplankton but also heterotrophs such as prokaryotes and flagellates.

### Conflict of interest statement

The authors declare that the research was conducted in the absence of any commercial or financial relationships that could be construed as a potential conflict of interest.
